# Ambient bright light treatment improved proxy-rated sleep but not sleep measured by actigraphy in nursing home patients with dementia: a placebo-controlled randomised trial

**DOI:** 10.1186/s12877-021-02236-4

**Published:** 2021-05-17

**Authors:** Gunnhild J. Hjetland, Eirin Kolberg, Ståle Pallesen, Eirunn Thun, Inger Hilde Nordhus, Bjørn Bjorvatn, Elisabeth Flo-Groeneboom

**Affiliations:** 1 Department of Clinical Psychology, Faculty of Psychology, University of Bergen, Bergen, Norway; 2 City Department of Health and Care, City of Bergen, Norway; 3 Norwegian Institute of Public Health, Bergen, Norway; 4 Norwegian Competence Center for Sleep Disorders, Haukeland University Hospital, Bergen, Norway; 5 Department of Psychosocial Science, Faculty of Psychology, University of Bergen, Bergen, Norway; 6 Department of Behavioural Medicine, Faculty of Medicine, University of Oslo, Oslo, Norway; 7 Department of Global Public Health and Primary Care, Faculty of Medicine, University of Bergen, Bergen, Norway

**Keywords:** Sleep, Dementia, Nursing home, Actigraphy, Sleep disorder inventory

## Abstract

**Background:**

Up to 70% of nursing home patients with dementia suffer from sleep problems. Light is the main zeitgeber to the circadian system and thus has a fundamental impact on sleep-wake behaviour. Low indoor light levels in nursing homes have been reported, and in combination with age-related reductions in light sensitivity, insufficient light exposure is likely to contribute to sleep problems in this population. Increasing daytime light exposure using bright light treatment (BLT) may represent a feasible non-pharmacological treatment for sleep problems in nursing home patients with dementia.

**Methods:**

The present study reports on sleep outcomes, which are the primary outcomes of the DEM.LIGHT trial (Therapy Light Rooms for Nursing Home Patients with Dementia– Designing Diurnal Conditions for Improved Sleep, Mood and Behavioural Problems), a 24-week cluster-randomised placebo-controlled trial including 8 nursing home units and 69 resident patients. The intervention comprised ambient light of 1000 lx and 6000 K from 10:00 to 15:00, with gradually increasing and decreasing light levels prior to and following this interval, using ceiling mounted light-fixtures and light emitting diode technology. The placebo condition had continuous standard light levels (150–300 lx, ~ 3000 K). Sleep was assessed at baseline and follow-up at week 8, 16, and 24, using the proxy-rated Sleep Disorder Inventory (SDI) and actigraphy (*Actiwatch II, Philips Respironics*). Mixed linear models were used to evaluate intervention effects, adjusting for relevant covariates such as age, gender, number of drugs, severity of dementia, eye disease, and estimated light exposure.

**Results:**

Sleep as measured by the SDI was significantly improved in the intervention group compared to the control group from baseline to week 16 (B = − 0.06, 95% CI -0.11 - -0.01, *p* < .05) and from baseline to week 24 (B = − 0.05, 95% CI -0.10 - -0.01, *p* < .05). There was no effect according to the SDI at week 8 and no significant effects in terms of actigraphically measured sleep.

**Conclusions:**

Proxy-rated sleep improved among nursing home patients with dementia following 16 and 24 weeks of BLT. These improvements were not corroborated by actigraphy recordings.

**Trial registration:**

ClinicalTrials.gov Identifier: NCT03357328. Registered 29 November 2017 – Retrospectively registered.

**Supplementary Information:**

The online version contains supplementary material available at 10.1186/s12877-021-02236-4.

## Background

Dementia denotes a group of disorders characterised by progressive neurodegenerative and/or vascular damage, with accompanying impairments of cognition and disturbances of mood and behaviour [1]. These disorders constitute a severe burden at the individual and societal level [2], making them a social health priority of the World Health Organization [3].

Disrupted sleep constitutes a major challenge in relation to dementia, as it affects up to 70% of the patients [4]. As sleep is essential for normal functioning, its disruption may have severe negative effects on cognitive, emotional, and physical functioning [5]. In the context of cognitive impairment, sleep disruption places a significant burden on caregivers [6]. Related events such as night-time wandering and confusion may increase the risk of falls and injuries [7, 8]. Importantly, sleep problems are associated with increased depressive symptoms [9–11], reduced functional status (ability to perform normal daily activities) [12], and cognitive decline [13]. Due to limited staff in nursing homes, pharmacological treatment (e.g., antidepressants, benzodiazepines, z-hypnotics, and antipsychotics) are often resorted to for relieving sleep disturbances [14]. Medications are, however, often associated with side-effects such as sedation and risk of falling [14, 15], and there is generally limited evidence for their efficacy in these populations [16]. Thus, identifying safe and effective treatments for disturbed sleep is of crucial importance.

As dementia progresses, brain systems involved in sleep-wake regulation are increasingly affected [5, 17]. Importantly, this includes neurodegeneration of the suprachiasmatic nucleus (SCN) of the hypothalamus, the main circadian pacemaker, which generates 24-h rhythms in hormone levels, body temperature, and sleep-wake behaviour. As a result, people with dementia often display a fragmented sleep pattern with several sleep and wake periods occurring throughout the 24-h day [17, 18]. Disrupted sleep may be further worsened by factors such as reduced social interaction, inactivity, and medications [19, 20]. In addition, light exposure plays a major role in circadian regulation, and insufficient exposure has consequently been associated with sleep problems in dementia patients [21, 22].

Light is detected by the intrinsically photosensitive retinal ganglion cells of the eye (ipRGCs; neurons in the retina containing the light sensitive photopigment melanopsin) and relayed directly to the SCN. The effects of light exposure on the circadian system depend on the timing, duration, illumination level (lux), and spectral composition (i.e., the colour of the light), as well as light exposure history [23]. The ipRGCs are maximally sensitive to short wavelengths (~ 480 nm) [24, 25], hence polychromatic light with high amounts of short wavelengths, i.e., blue light, therefore elicits stronger circadian responses than light with large amounts of long wavelengths (red-yellow) [26, 27]. The colour appearance of light is defined by correlated colour temperature (CCT), measured in Kelvin (K). Daylight has a large amount of blue light, with a CCT of about 5700–7700 K, depending on atmospheric conditions [28]. In comparison, standard light bulbs typically deliver more yellow light, with lower CCT (2700–3000 K).

With increasing age, lens yellowing and excessive pupil constriction (senescent miosis) reduce the amount of light reaching the retina [29, 30]. Although some evidence has suggested compensatory mechanisms preserving light sensitivity [31], lens yellowing has been associated with self-reported sleep disturbances [32]. Further, Alzheimer’s disease is associated with several pathological changes in the visual system, including loss of ipRGCs [33]. Several studies have reported light levels in nursing homes far below what is considered necessary for circadian entrainment (synchronising of an organisms’ rhythms to recurring environmental cues) [17, 34–37]. For example, in a study of seven nursing homes in the Netherlands, Sinoo et al. [37] found that 65–96% of the light measurements fell below 750 lx. In a recent study including 15 Norwegian dementia unit living rooms [38], median vertical illumination was below 300 lx, even in summer. In contrast, daylight ranges from 6500 to 130,000 lx depending on weather conditions [39, 40]. In sum, age and dementia-related changes to the eye and low indoor illumination in relevant institutions suggest that dementia patients are rarely exposed to light levels sufficient to entrain the circadian clock. Therefore, increasing light exposure, i.e., bright light treatment (BLT), constitutes a promising non-pharmacological treatment for disrupted sleep in people with dementia.

Traditionally, BLT has been administered using light boxes with high illumination white light (2500–10,000 lx at distances from 10 to 50 cm) for 30 to 120 min each day. This requires the patient to sit relatively still and face the box. Several studies have reported within-group improvements of sleep outcomes using this type of BLT [41–47], although the results are not consistent [48–50]. An issue with light boxes is that dementia patients are in need of continuous supervision to receive sufficient light exposure [42], which is often too demanding for the staff. Recent advances in light emitting diode (LED) technology have allowed for the manipulation of both light illumination and its spectral composition. This technology is able to deliver light of high CCT and illumination and may be used to change the ambient light of entire rooms. Researchers have in this realm typically used light ranging from 6500 K and 1200 lx to 13,000 K and 400 lx [51]. By this way of administering light, the patients may move around freely, and the need for staff to ensure treatment adherence is eliminated. In addition, such systems may be programmed to provide light contrasts between midday (high illumination short wavelength light) and early mornings and evenings (lower illumination and “warmer”, longer wavelength light).

Although BLT has been evaluated as a treatment for sleep problems and emotional and behavioural symptoms in dementia populations for two decades, conclusive results about its efficacy are lacking. Recent meta-analyses, with 6 to 11 studies included, have reported only small effects (Hedges’ g of 0.25–0.30) of BLT in dementia [52, 53]. Generally, few randomised controlled trials have been conducted and there are considerable variations in methods and designs, such as the timing, duration, and means of delivering BLT. Hence, there is a need for more high-quality studies [51].

In the present study we report the primary outcomes from the DEM.LIGHT trial aiming to evaluate the effects of a ceiling-mounted dynamic ambient BLT solution on sleep in nursing home patients with severe dementia. We hypothesised that the BLT condition would improve sleep measured by actigraphy and a proxy-rated sleep scale across the 24-week treatment period, as compared to a control group receiving conventional light with standard and constant light levels.

## Methods

The present paper is based on data from the 24-week cluster randomised placebo-controlled trial “*Therapy Light Rooms for Nursing Home Patients with Dementia– Designing Diurnal Conditions for Improved Sleep, Mood and Behavioural Problems*”, the DEM.LIGHT trial (ClinicalTrials.gov Identifier: NCT03357328). This trial evaluated the effect of a BLT solution on sleep, circadian rhythmicity, mood, behaviour, and function in nursing home patients with dementia. The present paper reports on the sleep outcomes of the DEM.LIGHT trial. The other outcomes will be reported in subsequent papers. The DEM.LIGHT trial was conducted from late September 2017 to early April 2018, in Bergen, Norway (60°3’N, 5°3’E). The intervention period lasted for 24 weeks and data were collected at baseline, week 8, week 16, and week 24. The present study adheres to the CONSORT guidelines [54].

### Participants

The Department of Health and Care, City of Bergen, Norway, supported the study by providing information about eligible nursing home dementia units (i.e., nursing homes not involved in other trials or quality of care projects, and where ceiling light instalment was possible). The management of eligible nursing homes was contacted by the two researchers leading the data collection (GJH, EK). Site visits were conducted to provide information about the study and assess if light instalment was possible. In total, 14 nursing homes were approached, of which 8 units from eight nursing homes were included. Nursing home units and patients were recruited between September 2016 and August 2017. The eligibility criteria were discussed with the resident medical practitioner of each nursing home before all the eligible patients at the unit were invited to participate. Inclusion criteria were as follows: The patient had to be 60 years of age or older, in long-term care (> 4 weeks), have dementia in accordance with DSM-5 criteria and either sleep/circadian rhythm disturbances, BPSD, or severely reduced activities of daily living. Exclusion criteria were blindness or other reasons the patient was unable to benefit from light; the patient was taking part in another trial; a condition that contra-indicated the intervention; an advanced, severe medical condition and/or expected survival of less than 6 months; psychosis or a severe mental disorder; or other aspects that could interfere with participation. Due to the long follow-up of 6 months, it was not possible to maintain stable doses of medications during the course of the study. The number of psychotropic medications were, however, controlled for in the analyses.

### Procedures

#### The intervention

**Fig. 1:**
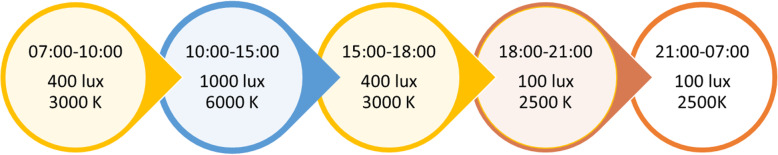
An illustration of the intervention light sequence. The light changed gradually across 30 min between each condition. Each nursing home could choose if they wanted to turn the lights off or to maintain 100 lx and 2500 K throughout the night (21:00–07:00). The patients generally did not spend time in the common room during the night, and whether or not the light was on in the common room depended on the needs of the staff during the night

A LED ceiling-mounted bright light solution, delivered by Glamox AS (supplier of professional lighting solutions), was installed in the common rooms of the four intervention units. Glamox engineers calculated the number of LED units needed in each common room (Glamox, 1 x C95 48 CCT 6500 K MP 47 W / 4702 lm), accounting for the number and direction of windows. The light panels were programmed to provide a cycle of gradually higher illumination and CCT during the day and then lower illumination and CCT in the evening (see Fig. 1), with the highest light levels of 1000 vertical lux and 6000 K from 10:00 to 15:00. The light equipment was installed prior to the start of the study and light levels were maintained at standard levels (approximately 100 lx and 3000 K) before the study commenced so that staff and participants were familiarised with the new light units. The intervention light sequence was activated following baseline data collection. The control panel for the light was locked with a personal identification number only known to the researchers. The contact information of the researchers was provided in case of any problems with the light setup.

The placebo control condition was created by replacing the light bulbs in the existing fittings with conventional 3000 K light bulbs in all common rooms (CFL AURA UNIQUE-D/E LL 18 W/830 G241–2 in three common rooms and CFL AURA UNIQUE-L LL 18 W/830 2G11 in one), thus largely maintaining the pre-existing light levels in these units. These light bulbs were installed immediately following the baseline data collection. After the intervention was activated and the placebo lights were installed, the daytime light levels were measured by the researchers in all eight common rooms using the *GL Spectis 1.0 T Flicker* spectrometer (*GL Optic*). Spectral analysis was performed in *GL Spectrosoft*. Vertical measurements were recorded to approximate corneal illumination (120 cm above the floor), and horizontal measurements were recorded at the typical height of reading or other visual tasks (80 cm above the floor). Effective illuminances were calculated according to the α-opic illuminance model using the irradiance toolbox by Lucas et al. [55].

### Outcomes

All questionnaires used in the present study were completed by daytime nursing staff that knew the patients well, i.e., regular staff working directly with the patients. Research staff involved in the DEM.LIGHT trial guided the nurses in using the assessment tools. The questionnaires were completed once at each data collection time point: either the same week the patients wore the actigraphs or the following week. Data regarding sociodemographic characteristics, diagnoses, and medication status were collected from medical records. One of the researchers was granted access to extract these data from a centralised journal system used in nursing homes by Bergen Municipality.

#### Sleep outcomes

Changes in sleep parameters comprised the primary outcome of the DEM.LIGHT trial. Sleep was proxy-rated by the nursing staff using the Sleep Disorder Inventory (SDI) [12]. The SDI assesses seven symptoms that are scored in terms of frequency (0–4), severity (0–3), and caregiver distress (0–5). The SDI total score was calculated as the sum of the products of the frequency and severity ratings of each of item 1–7 (range: 0–84). The SDI has previously been demonstrated to correspond well to actigraphy outcomes in this population [56], as well as in home-dwelling dementia populations [12]. A total score of five or more indicates the presence of clinically significant sleep disruption [56].

Actigraphs (*Actiwatch II, Philips Respironics)* were used to provide an objective estimation of sleep. Actigraphs are wrist-worn devices that measure activity continuously over days or weeks [57]. In accordance with previous studies on nursing home patients, the actigraphs were placed on the dominant or most mobile wrist [58–61] as this increases the chances of detecting movement in patients who are often immobile or lethargic. Based on the activity data, each epoch was scored as sleep or wake by the *Actiware software, version 6.0.9 (Philips Respironics)*. The threshold for wakefulness was set to medium and epoch length to 1 min, as recommended by Camargos et al. [61].

Determining the true bedtime and wake-time in nursing home patients is challenging, as these patients frequently spend more than 12 h in bed (e.g., [58, 60]). We initially planned to score the rest intervals based on a predetermined scoring protocol; however, there were rarely clear indications of bedtime and wake time, and the nurses did not consistently press the event buttons. Therefore, a fixed rest interval was set from 22:00 to 06:00, in accordance with previous studies, which ensured capturing the main sleep episode of the vast majority of participants [22, 34, 48, 62]. A fixed daytime interval was also set from 10:00 to 18:00. This interval corresponds to other studies in dementia populations, and was chosen to avoid including periods when the participants were in bed [63]. Activity data were collected for seven consecutive days at baseline, and at follow-up after 8, 16, and 24 weeks. Patients had to have at least five nights of recordings to be included in the analyses. The following actigraph outputs were extracted: Sleep efficiency (SE; the percentage of time spent asleep in the rest interval), TST rest (total sleep time in the rest interval), 24-h TST, daytime TST (TST in the daytime interval), and wake-after-sleep-onset (WASO; the time spent awake after sleep onset) in the rest interval. Since TST rest and SE are perfect linear functions of each other in a fixed rest interval, including both in the analyses would be redundant and TST rest was only used as a descriptive variable.

#### Other outcomes

In addition to sociodemographic data, several secondary outcomes were used to describe the population at baseline and as control variables in the analyses. The Mini Mental State Examination (MMSE) assesses the level of cognitive impairment (range: 0–30), where a low score indicates worse cognitive function [64]. The Functional Assessment Staging (FAST) [65] rates the severity of dementia according to seven stages, where a score of 6–7 indicates severe dementia. The Charlson Comorbidity Index (CCI) assigns weights to 17 comorbidities to assess the burden of comorbid disease, where a higher score is associated with a higher mortality risk [66]. To assess activities of daily living (ADL), the instrument by Lawton and Brody was used [67]. This scale includes six items (composite score range 0–30), where a lower value indicates better functioning and independence. The nursing home version of the Neuropsychiatric Inventory (NPI-NH) [68, 69] is a proxy-rated tool that assesses “Behavioural and Psychological Symptoms of Dementia” by assigning frequency and severity ratings to 12 symptoms (delusion, hallucination, agitation, depression, anxiety, euphoria, apathy, disinhibition, irritability, aberrant motor behaviour, night-time behaviour and eating disturbance).

The staff at each unit were urged to report any change in patient health or behaviour in relation to the intervention. Potential adverse events and tolerability were also monitored at each data collection visit.

### Adherence to treatment

To assess adherence to treatment, a questionnaire was administered to the nursing staff, where they were asked to estimate the time each patient spent in the common room during different epochs of the day (average during the last 8 weeks). The epochs corresponded with the light cycle of the intervention, so that the day was split into 07:00–10:00, 10:00–15:00, 15:00–18:00, and “after 18:00”. The nursing staff provided a time estimate for each epoch in terms of hours and minutes. They were also instructed to report the number of days when the patient was not present in the common room, and informed that these days should not be included in the above-mentioned estimate. For the purpose of this study, the time estimates corresponding to the epoch with the highest light levels (10:00–15:00) were included in the analyses.

### Sample size and power

Expecting moderate effect sizes (Cohen’s d = .50) for the actigraphy outcomes, a .05 alpha level (two-tailed), and the power set to .80, the power-analysis showed that 64 participants from a minimum of 8 clusters were needed in order to detect differences between conditions. Expecting a 20% dropout, the aim was to recruit 80 participants [70, 71].

### Randomisation

After eight nursing home units were included, clusters (each unit constituted one cluster) were randomised to the intervention condition (1) or the control condition (0) using random group assignment in SPSS. Randomisation and enrolment were completed by the research group.

### Blinding

Although the placebo effect might not be an issue in those with severe dementia, staff might be affected, creating bias such as the Hawthorne effect [72, 73]. Hence, potential changes in staff routines and behaviour in response to the treatment may affect outcomes, making a control condition necessary. Because staff and participants could not be kept blind to the intervention, as the light setup comprised an obvious change in the common room, new light bulbs were fitted in the control units to mimic an intervention and ensure similar light levels across the control units. The participants and nursing staff were consequently unaware of condition assignment and all included units were located at different nursing homes to minimise threats to internal validity in terms of performance and detection bias [74]. After the end of the trial, the study design was explained to the staff, who were then asked to answer a questionnaire asking whether they thought they were in the control or intervention group.

### Statistical analyses

We initially planned to analyse data using ANOVA, but owing to some missing data due to non-compliance to actigraphy and because some patients passed away or moved to another facility during the study, we evaluated the intervention effects by means of linear mixed models using the lme4 package in *R* [75, 76]. This analysis method takes into account all available data, performs better than alternatives when there are missing data, and estimates fixed effects while adjusting for correlation due to repeated measurements on each subject [77, 78]. Linear mixed models using restricted maximum likelihood estimation were used to assess all outcomes. Group (intervention vs. control), time (treated as categorical with levels baseline, 8 weeks, 16 weeks, and 24 weeks), and the group-by-time interaction were included as fixed effects in the model. The models were fitted with random intercepts at the patient level to account for intra-participant correlation of the outcomes, and random slope was included if model fit improved. Models were selected based on best fit according to likelihood ratio tests.

A range of factors are likely to impact the response to BLT, and a range of predetermined prognostic variables were tested as covariates. AIC and BIC were examined, and likelihood ratio tests were performed using the Anova package in *R*. Furthermore, covariates that improved fit were added to the model. The following list of covariates were tested: Age, gender, number of psychotropic medications, CCI-scores, FAST-scores, MMSE-scores, eye disease, whether the patient passed away or moved during the study (drop-out), and average time in the common room during the day (between 10:00 and 15:00). Melanopic lux was also tested as a covariate because the light levels varied within the intervention group (ranging from 675 to 1050 mean vertical melanopic lux) and control group (56 to 261 melanopic lux) (Table S1).

Preliminary analyses showed that the SDI data were highly skewed, consequently the scores were transformed to achieve normal distribution by adding a constant of 0.5 and using a Box Cox transformation, which resulted in a lambda of 0.6.

Further, the relationship between sleep, demographic and secondary outcomes at baseline was investigated by using correlation analysis for continuous data and comparing groups for categorical data using Student’s t-test. Differences in light levels between the intervention and the placebo condition, as well as differences in light exposure time were evaluated using Student’s t-tests and Mann-Whitney U tests in SPSS for Windows, version 25 (IBM).

### Missing data

If single questionnaires were missing data on 20% or more of items on the SDI, they were excluded from the analyses. For questionnaires missing less than 20%, and where data were missing completely at random, imputations were made using expectation maximisation (EM) in SPSS*.*

## Results

**Fig. 2:**
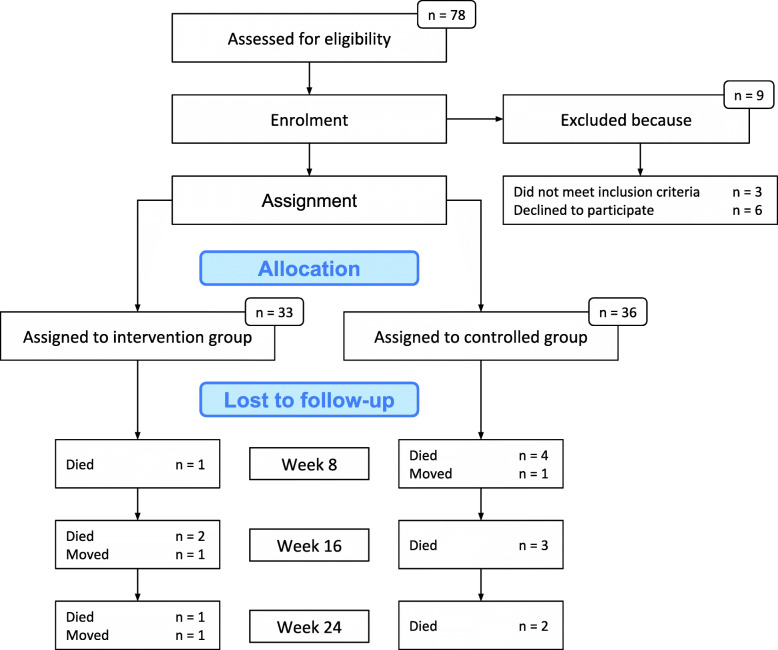
Showing the flow of participants from eligibility assessment to study completion. Note: Main cause for moving was a deterioration of somatic health requiring the patient to live in a somatic ward

**Fig. 3:**
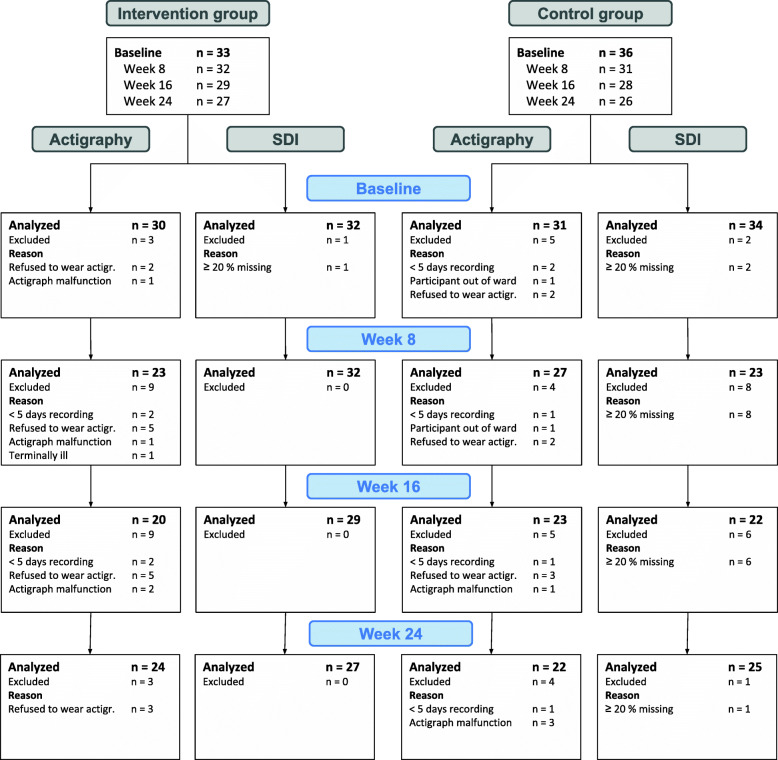
Overview of missing data and reason for missing at each data collection time point, not including those lost to follow-up

Seventy-eight patients were evaluated for eligibility, of which 69 were recruited to the study (see the flow chart in Fig. 2). Figure 3 shows the number of participants included in each analysis at each time point and reasons for missing data (not counting data missing due to death or moving away). Of the 69 patients who were enrolled, the mean age was 84 years, and 68% were female. The mean MMSE score was 6.4 (SD = 6.7) (Table 1).

**Table 1:** Descriptive statistics at baseline for the 69 patients

	Control group (*n* = 36)	Intervention group (*n* = 33)	Whole group (*n* = 69)
Age (mean, SD)	82.8 (7.9)	84.3 (6.2)	83.5 (7.1)
Female (%)	61	76	68
MMSE (*n* = 56), median (25th–75th percentile)	3.0 (1.0–7.0)	6.0 (2.0–10.0)	4.0 (1.0–9.5)
Dementia diagnoses, *n* (%)			
AD	20 (55.6)	18 (54.5)	38 (55.0)
VD	2 (5.6)	2 (6.0)	4 (6.0)
Mixed AD and VD	0	0	0
LBD	1 (2.8)	0	1 (1.5)
FTD	0	0	0
PD	0	0	0
Unknown dementia	10 (27.8)	11 (33.3)	21 (30.5)
Other dementia	1 (2.8)	1 (3.0)	2 (3.0)
No diagnosis^*^	2 (5.6)	1 (3.0)	3 (4.0)
NPI-NH total score (*n* = 69), median (25th–75th percentile)	12.5 (5.5–42.5)	24.0 (11.0–42.0)	21.0 (6.0–42.0)
ADL (*n* = 69) (mean, SD)	18.4 (4.7)	17.1 (5.1)	17.8 (4.9)
FAST (*n* = 67), median (25th–75th percentile)	6.0 (6.0–7.0)	6.0 (6.0–6.0)	6.0 (6.0–6.0)
CCI (*n* = 69), median (25th–75th percentile)	1.0 (1.0–2.0)	2.0 (1.0–2.0)	1.0 (1.0–2.0)
No. of drugs (mean, SD)	6.7 (3.0)	6.5 (2.7)	6.7 (2.8)
No. of psychotropic drugs^¤^, median (25th–75th percentile)	3.0 (1.3–3.8)	3.0 (2.0–4.0)	3.0 (2.0–4.0)
Any psychotropic, *n* (%)	34 (94%)	31 (94%)	65 (94%)
No. of sedatives^§^, median (25th–75th percentile)	0.0 (0.0–0.0)	0.0 (0.0–0.0)	0.0 (0.0–0.0)
Any sedatives, *n* (%)	3 (8%)	6 (18%)	9 (13%)

*AD* Alzheimer’s Disease, *ADL* Activities of Daily Living, *CCI* Charlson Comorbidity Index, *FAST* Functional Assessment Staging, *FTD* Frontotemporal dementia, *LBD* Lewy Body dementia, *MMSE* Mini Mental State Examiner, *NPI-NH* Neuropsychiatric inventory – nursing home version, *PD* Parkinson’s dementia, *VD* Vascular dementia

Note: Medians and the 25th and 75th percentiles are presented for non-normal data. Differences at baseline were evaluated using the Student’s t-test for parametric data and the Mann-Whitney U test for non-parametric data (marked in italics). The Chi-square probability distribution was used to analyse categorical data

*These patients were still included as their scores on the Mini Mental State Examination and the Functional Assessment Staging suggested moderate and severe dementia. In addition, clinically trained researchers concluded that they with high probability suffered from dementia according to the DSM-5 criteria

^**¤**^Psychotropic drugs include all drugs coded as N in the ATC system

^§^Sedatives include all N05C drugs, including z-hypnotics

Table 2 shows the mean light levels in the intervention and control condition, respectively. All light metrics were significantly higher in the intervention group than the control group (all *p*’s < .05 and Bonferroni adjusted *p* < .008). The supplemental Table S1 shows the light measurements separately for each unit. There was some variability in light levels across intervention units, and one unit in particular had lower than intended vertical illumination (722; SD = 69; photopic lux and 675; SD = 62; melanopic lux). Also, one control unit had higher illumination than the other control units, with 368 (SD = 99) photopic lux and 261 (SD = 125) melanopic lux. All other control units had between 134 and 271 photopic lux and between 56 and 143 melanopic lux. Both horizontal and vertical measurements and photopic lux, melanopic lux, and CCT values are provided in Table 2 as well as in supplementary Table S1. Across the intervention period, participants in the intervention group spent a mean of 3 h and 24 min (SD = 1 h 30 min), and the control group spent 3 h and 0 min (SD = 1 h 42 min) in the common room during the time of day with the highest light levels (10:00–15:00), as estimated by staff (Table 3). There were no significant differences between the groups regarding time spent in the common room at any time point.

**Table 2:** The light levels in the intervention group and the control group

	Mean (SD; min-max) vertical photopic lux	Mean (SD; min-max) vertical melanopic lux	Mean (SD; min-max) horizontal photopic lux	Mean (SD; min-max) horizontal melanopic lux	Mean (SD; min max) Kelvin vertical	Mean (SD; min-max) Kelvin horizontal
Intervention	1039 (225; 722–1242)	915 (174; 676–1050)	1842 (528; 1388–2585)	1680 (557; 1270–2488)	5369 (251; 5088–5641)	5521 (275; 5211–5879)
Control	242 (102; 134–368)	137 (90; 56–261)	343 (65; 259–408)	178 (52; 106–218)	3049 (470; 2707–3622)	3048 (397; 2707–3048)
*p*-value	.001	.000	.001	.002	.000	.000

Note: Vertical lux was measured 120 cm above the floor in the middle of the room in four directions that were averaged to produce the mean vertical illumination. Mean horizontal lux was calculated as the mean of measurements 80 cm above the floor in the middle of the room and 50 cm from the back wall. Mean lux values were calculated for the intervention and control group by averaging the values across clusters. Differences between groups tested with Student’s t-test

**Table 3:** The median time spent in the living room between 10:00 and 15:00 for each group

	Week 8		Week 16		Week 24	
	Mean (SD)	Median (25th–75th)	Mean (SD)	Median (25th–75th)	Mean (SD)	Median (25th–75th)
Intervention	3.2 (1.8)	3.3 (1.8–5.0)	3.4 (1.8)	4.0 (2.0–5.0)	3.7 (1.6)	5.0 (2.0–5.0)
Control	3.1 (1.3)	3.0 (2.0–4.0)	2.9 (1.6)	3.0 (1.5–5.0)	2.9 (1.7)	3.0 (2.0–5.0)
*p*-value	.560		.482		.077	

Note: Differences between groups tested with Mann-Whitney U test. Time in hours

The primary outcome of this randomised controlled trial (RCT) was sleep. The linear mixed model analyses for actigraphically measured sleep outcomes showed no statistically significant differences between patients who received BLT compared to those who received standard light (see Table 4).

**Table 4:** Showing the results of the linear mixed model analyses

Variables	***n***	Nmb obs	Baseline to week 8				Baseline to week 16				Baseline to week 24			
			Regression coefficient	Std.error	***P***	CI	Regression coefficient	Std.error	***P***	CI	Regression coefficient	Std.error	***P***	CI
SE^a^	65	201	−0.32	3.18	.920	−6.56-5.92	2.97	3.36	.377	−3.60-9.55	−3.65	3.26	.264	−10.04-2.73
TST 24 h^b,c^	65	194	−52.18	30.86	.093	−112.66-8.03	−36.11	33.95	.290	−102.64-30.43	−12.46	31.41	.692	−74.03-49.10
TST day^c^	65	201	6.65	14.03	.636	−20.84-34.14	24.83	14.79	.096	−4.16-53.81	21.44	14.35	.138	-6.69-49.57
WASO^d^	65	201	14.42	10.43	.169	−6.03-34.86	2.59	10.98	.814	−18.92-24.11	20.92	10.68	.052	0.01–41.85
SDI trans^e^	66	213	−0.04	.02	.094	−0.09-0.01	−0.06	.03	**.020**	−0.11--0.01	− 0.05	0.02	**.028**	−0.10--0.01

Note: The R package lme4 assumes an unstructured variance-covariance structure

*CI* confidence interval, *Nmb obs* Number of observations, *P* p-value, *SDI* Sleep Disorder Inventory, *SE* (variable) sleep efficiency, *std.error* standard error, *trans* transformed, *TST* total sleep time, *WASO* wake after sleep onset

Note: The regression coefficients reflect the change from baseline in the intervention group compared to the control group

^a^Eye disease included as covariate

^b^Drop-out included as covariate

^c^Total Functional Assessment Staging score in the relevant week included as covariate

^d^Age included as covariate

^e^Average number of psychotropic medications included as covariate

For the SDI, the regression coefficient for the time*group interaction, representing the group difference in change from baseline, was −.06 (95% CI −.11 to −.01; *p* = .020) at week 16 and − .05 (95% CI −.10 to −.01; *p* = .028) at week 24, indicating relatively better sleep in the intervention group (i.e., less disrupted sleep) than the control group at these time points. The average number of psychotropic drugs was included as a covariate in this model. There was no significant effect at week 8 (Table 4).

Table 5 shows the means and standard deviations for the intervention group and the control group for each outcome at each time point, as well as medians and 25th and 75th percentiles for non-normal data.

**Table 5:** Observed means and standard deviations for the groups at each time point, with medians and 25th and 76th percentiles reported for non-normal data

		Intervention group				Control group			
		Baseline	Week 8	Week 16	Week 24	Baseline	Week 8	Week 16	Week 24
SE	Mean (SD)	75.0 (18.6)	74.8 (14.5)	76.5 (15.7)	71.8 (18.8)	72.4 (16.3)	70.3 (15.4)	69.2 (13.4)	71.7 (15.2)
	Median (25th–75th)	80.2 (67.0–90.8)	76.9 (67.7–88.5)	80.0 (70.7–89.1)	76.6 (58.1–87.0)	79.0 (59.0–87.5)	75.7 (55.3–81.3)	70.2 (60.7–79.4)	72.4 (60.5–84.2)
TST night	Mean (SD)	359.9 (89.3)	358.8 (69.6)	367.0 (75.3)	344.5 (90.0)	347.6 (78.3)	339.2 (75.2)	332.0 (64.3)	344.1 (73.2)
	Median (25th–75th)	385.1 (321.4–432.1)	368.3 (324.9–425.0)	383.4 (240.0–427.7)	367.6 (278.6–415.2)	379.1 (283.1–419.9)	364.4 (265.6–394.7)	336.9 (291.3–381.3)	347.4 (290.6–404.3)
TST 24 h	Mean (SD)	623.3 (162.0)	623.3 (162.0)	597.7 (137.3)	626.2 (179.7)	669.2 (182.7)	655.5 (157.1)	646.2 (152.9)	664.6 (186.4)
TST day	Mean (SD)	125.4 (70.3)	119.9 (73.0)	141.2 (100.2)	137.1 (82.3)	176.2 (93.1)	150.0 (91.5)	159.0 (90.9)	154.5 (88.6)
WASO	Mean (SD)	58.8 (32.4)	65.7 (36.4)	56.3 (23.7)	75.3 (48.7)	76.3 (50.0)	73.4 (43.5)	73.6 (33.7)	71.7 (39.1)
SDI	Mean (SD)	10.3 (14.9)	8.0 (8.8)	7.7 (12.3)	6.9 (10.7)	10.0 (12.8)	19.2 (21.3)	10.8 (10.5)	9.4 (10.3)
	Median (25th–75th)	3.0 (0.0–13.0)	5.5 (1.5–12.0)	2.0 (0.0–12.0)	2.0 (0.0–10.0)	3.0 (0.0–18.0)	12.0 (2.0–32.0)	5.5 (2.0–21.0)	5.0 (3.0–14.0)
	Proportion with sleep problems, n (%)*	12 (40.0)	11 (47.8)	8 (40.0)	8 (33.3)	14 (45.2)	12 (44.4)	14 (60.9)	12 (54.5)

*Sleep problems defined as sleep efficiency < 75%

*SE* sleep efficiency, *SD* standard deviation, *SDI* Sleep Disorder Inventory, *TST* total sleep time, *WASO* wake after sleep onset

At baseline, none of the actigraphy sleep parameters correlated with age, total number of drugs, number of psychotropic drugs, number of sedatives, cognitive impairment, dementia severity, or comorbidity (Table 6). The SDI score had significant positive correlations with the total number of medications (Spearman’s rho = .30, *p* = .015) and number of psychotropic medications (Spearman’s rho = .35, *p* = .004). There were no gender differences in sleep outcomes at baseline, in either WASO (*p* = .419), SE (*p* = .622), TST day (*p* = .154), TST 24 h (*p* = .216), or SDI (*p* = .356). Patients with and without eye disease did not differ significantly on WASO (*p* = .167), TST day (*p* = .792), TST 24 h (*p* = .098) or the SDI (*p* = .142). However, patients with eye disease had significantly lower SE at night (median of 57% vs. 80%, *p* = .003) than those without eye disease.

**Table 6:** Showing the correlation coefficients (*p*-values) between the sleep variables and scores on other variables at baseline

	Age	No. drugs	*No. psychotropic*	*No. sedatives*	*MMSE*	*FAST*	*CCI*
WASO	−.18	−.03	.04	−.06	−.03	.01	.03
TST day	−.01	−.01	.04	−.18	.04	−.05	−.05
TST 24 h	.02	.05	.09	−.15	.07	−.12	−.08
*SE*	.01	.01	.02	.01	.09	−.11	−.04
*SDI*	−.12	.30*	.35**	.19	−.03	−.05	.07

Note: The significance level was set to *p* < .05. Italics indicate non-normal data. Any pair with non-normal data was analysed using Spearman’s rho (italics). Normal data was analysed using Pearson correlation

*CCI* Charlson Comorbidity Index, *FAST* The Functional Assessment Staging, *MMSE* Mini Mental State Examination, *TST* total sleep time, *SDI* Sleep Disorder Inventory, *SE* sleep efficiency, *WASO* wake after sleep onset

*Significant at *p* < .05

**Significant at *p* < .01

No adverse events in relation to the intervention were reported by the staff. There was no significant deterioration of sleep outcomes in the intervention group compared to the control group. There were also no negative outcomes in terms of mortality or BPSD other than sleep (not published).

In the intervention group, 5 out of 19 respondents guessed that they were in the control group, and 2 out of 12 respondents in the control group thought that they were in the intervention group.

## Discussion

The present study found no evidence for effects on actigraphically measured sleep following 24 weeks of BLT. However, compared to the control group, the intervention group fared better from baseline to week 16 and from baseline to week 24, as measured by the proxy-rated Sleep Disorder Inventory (SDI). For the SDI, the observed means indicate that the median SDI scores for the control group deteriorated from baseline to week 8 and returned to baseline levels at week 16 and 24, while the intervention group had a slight improvement from baseline to week 24. These differences were significant at week 16 and 24 in the mixed linear model.

The non-significant findings of the intervention attested to by actigraph data are in line with results based on objective sleep data from some previous RCTs in nursing home populations [48, 49] and home-dwelling people with dementia [79]. However, other RCTs have shown beneficial effects of bright light on actigraphically assessed sleep parameters [45, 80, 81]. In a recent systematic review, we addressed several factors that may mediate the effects of BLT [51]. Although we endeavoured to reduce potential confounders in the present study, some issues related to the population, exposure, and assessment tools need to be taken into consideration. Firstly, the difference in outcomes depended on means of assessing sleep, with the analyses showing a significant treatment effect for the staff-rated SDI, in contrast to actigraphically assessed sleep. This is in line with the findings by Blytt et al. [58], who reported a significant discrepancy between actigraphically measured sleep and proxy-rated sleep in nursing home patients. In a recent publication, we found a satisfactory correspondence between the SDI and actigraphy data at baseline, in the same study population as the present study [56]. Still, actigraphy and the SDI are two very different measures of sleep, and it is possible that actigraphy was not responsive to change to the same degree as the SDI. Actigraphy is, at its core, a measure of activity, while the SDI comprises clinical assessments of sleep made by nursing home staff. Immobility during wakefulness may be miscoded as sleep by the actigraphs, and thus subtle changes in sleep and wakefulness may not be detected. Actigraphy has been shown to have a high sensitivity (ability to detect sleep) of 87–99%, albeit a low specificity (ability to detect wakefulness) of 28–67% compared to polysomnography, with lower specificity occurring when sleep efficiency is reduced [82]. In older adults with insomnia, Sivertsen et al. [83] found a specificity of 36%. A potentially low specificity, i.e., poor wake detection, could have impacted the outcomes of this study. Thus, actigraphy may not have been the optimal tool to detect wakefulness in this old, multimorbid and frail population. Hence, the field needs better and more accurate, yet feasible, objective sleep recordings in dementia. One promising tool in that regard is sleep radars, which are completely non-invasive and can collect data about whole body movement, including respiration [84]. However, sleep radars have not been validated for use in nursing homes or in people with dementia. The fact that the SDI-score correlated with medication use (total number and number of psychotropic drugs), may suggest that it was clinically relevant and identified clinically significant sleep symptoms in this population.

While using a fixed rest interval for the actigraphy data was considered the best solution in the present study, this comes with important caveats. Nursing home patients may spend a substantial amount of time in bed [58], and thus the main sleep episode may take place partially outside the chosen interval from 22:00 to 06:00. Importantly, shifting the timing of the main sleep episode may significantly impact the results, even though the person’s sleep is otherwise identical. For example, if a patient slept from 22:30–06:00 at baseline and then shifted the sleep episode to 23:30–07:00 at follow-up, this would according to the actigraph be interpreted as a deterioration of sleep. The SDI is more flexible in this regard.

Further, the somatic and mental health status of the patients may have affected sleep and/or mobility to the extent that it reduced discernible changes in the actigraphy-measured sleep. In addition, there was a high use of psychotropic drugs which combined to a high sedative load [85]. This may have reduced mobility and consequently affected the actigraphy data to a greater extent than the SDI data. In addition, somatic conditions including pain are known to reduce movement in this population [86].

The study participants were old (mean 84 years) and had severe dementia. As neurodegeneration increasingly affects sleep regulatory systems and eye physiology [5, 17, 29, 30], the lack of effects on actigraphy outcomes may indicate that the patients had limited benefit from BLT. Hence, it is possible that people with milder dementia would have benefitted more from BLT. This should be addressed in future studies.

Another important aspect to consider is the presence of eye disease among the participants. At baseline, those with eye disease had worse sleep than those with no eye disease. This variable was, however, only included in the final regression model of SE, as it affected the model fit only for this outcome, indicating that the presence of eye disease did not significantly influence the outcomes. Still, the difference at baseline may suggest that the baseline lighting was more problematic to those with impaired vision; however, there were too few individuals to investigate whether this group in particular benefitted from BLT.

Environmental factors may have confounded intervention effects. One of the first studies using ceiling-mounted light found that morning and all-day treatment (6500 K and 500–600 lx) were associated with the greatest amount of sleep, compared to evening treatment and standard light [87]. However, when controlling for study location, they found opposing effects of BLT on daytime sleepiness in the two included nursing home units, suggesting that other factors were at play. In the nursing home context, the day-to-day routines have a fundamental impact on the patients. For example, patients are often put to bed quite early and helped out of bed quite late in the morning. Consequently, some patients may end up spending more than 12 h in bed [58]. This may cause low sleep efficiency and increase the risk of nocturnal wakefulness. Also, other factors such as noise and light exposure at night are known to contribute to disrupted sleep in nursing home patients [88]. Together, influences from such environmental factors might have attenuated any positive effects of BLT in the present study.

Because BLT only affected subjectively measured sleep, it should be considered if this was caused by placebo-by-proxy [89] or similar phenomena. Light is inherently visible, and it was thus impossible to create a placebo condition that was identical to the intervention. Consequently, it is possible that the nurses in the control group realised that the new light bulbs in fact delivered standard light levels and that placebo-by-proxy effects only operated in the intervention group. However, if such effects were present, they should have appeared already in week 8. Thus, it is likely that the discrepancy in results between the two approaches of measuring sleep relates to the inherent differences between these measures and not to placebo-by-proxy effects.

### Limitations

The present study had some important limitations. The results show that blinding was successful only for a minority of the staff. However, success of blinding was assessed after revealing the design to the staff, which has been argued to yield questionable/invalid data [54]. In addition, the response rate among the nurses was low, and it is possible that those who did answer the blinding questions were more interested in the research than the nurses who did not respond.

The exact amount of light received by each participant was not measured. Although equipment for the purpose of continuously measuring light exposure at eye level (e.g., [90]) has been developed (attaching a light meter to the head), this has limited feasibility in a dementia sample. Exposure estimations based on standardised light meter recordings and time spent in the common room as reported by the nurses were thus considered the best option, although it would not correlate perfectly with real exposure.

There was some variation in light levels across units, where one control unit had higher mean light levels (261 melanopic lux) compared to the other control units (56–143 melanopic lux), and one intervention unit achieved lower-than-intended light levels (675 melanopic lux compared to 900–1050) (Table S1). Still, there was a minimum of a 400 melanopic lux difference between the control unit with the highest light levels, and the intervention unit with the lowest light levels, and controlling for light levels did not impact the results.

In the present study, we implemented the intervention only during the day in the common rooms. Hence, bedroom lighting was not a part of the intervention. Light exposure during the night (e.g., in bedrooms) may significantly disrupt sleep and ensuring darkness or blue-depleted light in the patients’ bedrooms at night could perhaps have caused a more robust effect.

The timing of the study (September–April) was chosen to include the dark season. Even though indoor light levels in nursing homes are low even in sunny weather conditions [36], there is a theoretical possibility that any beneficial effects of daylight availability during the summer and early autumn lingered during the baseline data collection. Similarly, the last data collection at week 24 was partially performed after the spring equinox, which might represent a confounding variable.

Due to a higher attrition rate than anticipated and some missing data due to non-compliance to actigraphy (Fig. 3), we ended up with a lower number of participants than recommended by our power analysis. This needs to be taken into consideration when interpreting the results.

### Strengths

To avoid any potential carry-over effects of BLT, we used a parallel group design. The BLT was delivered for a long period of time to evaluate both short-term and long-term effects. Light levels were measured in a standardised manner and reported in the appropriate light metric accounting for the sensitivity of the circadian system (melanopic lux). This study demonstrated that it is feasible to install ambient light systems in nursing homes. Further, including dementia units from eight different nursing homes and using lenient inclusion criteria provided a representative study population and high ecological validity.

## Conclusion

The present findings indicate that BLT improved proxy-rated sleep among nursing home patients with dementia after 16 and 24 weeks of treatment compared to a control group. The lack of improvement in proxy-rated sleep at week 8 may suggest that several weeks of exposure is necessary to detect beneficial effects of BLT. There was no evidence for an effect of BLT on actigraphically measured sleep. Despite the results being mixed, there is reason to assume that BLT may be beneficial for people with severe dementia.

## Supplementary Information

**Additional file 1: Supplementary Table S1**. Table showing the light levels in each cluster of the intervention group and the control group

## Data Availability

The dataset analysed during the current study is available from the corresponding author on reasonable request.

## Abbreviations

ADL: Activities of Daily Living
BLT: Bright light treatment
CCI: Charlson Comorbidity Index
CCT: Correlated colour temperature
FAST: Functional Assessment Staging
ipRGCs: Intrinsically photosensitive retinal ganglion cells
K: Kelvin
LED: Light emitting diode
MMSE: Mini Mental State Examination
RCT: Randomised controlled trial
SCN: Suprachiasmatic nucleus
SDI: Sleep Disorder Inventory
SE: Sleep efficiency
TST: Total sleep time
WASO: Wake-after-sleep-onset
