# Supplementary material for: Ambient bright light treatment improved proxy-rated sleep but not sleep measured by actigraphy in nursing home patients with dementia: a placebo-controlled randomised trial
Source: BMC Geriatr. 2021 May 17;21:312. doi: 10.1186/s12877-021-02236-4 (PMC8127192; doi:10.1186/s12877-021-02236-4)
Supplement: Supplementary file 1 — Additional file 1: Supplementary Table S1. Table showing the light levels in each cluster of the intervention group and the control group [file 12877_2021_2236_MOESM1_ESM.docx]

Table S1: Showing the mean (SD; range) lux and Kelvin of each cluster in the intervention group (BLT 1-BLT 4) and the control group (Control 1-Control 4).

|  | Mean vertical, photopic lux | Mean vertical, melanopic lux | Mean horizontal, photopic lux | Mean horizontal, melanopic lux | Mean vertical, Kelvin |
| --- | --- | --- | --- | --- | --- |
| BLT 1 | 1138 (168; 1005-1382) | 1037 (189; 891-1305) | 2585 (142; 2485-2685) | 2488 (291; 2282-2693) | 5507 (215; 5230-5723) |
| BLT 2 | 722 (69; 641-796) | 675 (62; 602-749) | 1388 (261; 1203-1572) | 1270 (268; 1080-1460) | 5641 (113; 5478-5723) |
| BLT 3 | 1252 (122; 1170-1424) | 1050 (133; 973-1248) | 1566 (888; 938-2194) | 1358 (836; 767-1949) | 5088 (108; 5001-5245) |
| BLT 4 | 1055 (101; 952-1181) | 900 (78; 829-994) | 1829 (452; 1509-2148) | 1604 (421; 1307-1902) | 5240 (81; 5163-5313) |
| Control 1 | 271 (40; 217-312) | 143 (23; 113-162) | 408 (1,31; 497-409) | 218 (5; 214-222) | 2917 (100; 2827-3059) |
| Control 2 | 134 (26; 105-163) | 56 (12; 42-68) | 259 (156; 148-369) | 106 (69; 57-154) | 2705 (72; 2615-2777) |
| Control 3 | 368 (99; 274-507) | 261 (125; 148-439) | 328 (116; 246-410) | 215 (62; 172-259) | 3743 (617; 3165-4604) |
| Control 4 | 193 (18; 170-208) | 88 (9; 76-95) | 376 (121; 290-462) | 171 (57; 131-211) | 2832 (40; 2796-2880) |

BLT= Bright light treatment

Note: Vertical lux was measured 120 above the floor in the middle of the room in four directions; towards the window, towards the right, the left, and towards the back of the room. These four values were averaged to produce the mean vertical illumination. Horizontal lux was measured in the middle of the room, at 80 cm above the floor, as well as 50 cm from the window and 50 cm from the back wall. Mean horizontal lux was calculated as the mean of the measurements in the middle of the room and by the back wall.
